# A case with concurrent duplication, triplication, and uniparental isodisomy at 1q42.12-qter supporting microhomology-mediated break-induced replication model for replicative rearrangements

**DOI:** 10.1186/s13039-017-0316-6

**Published:** 2017-04-28

**Authors:** Tomohiro Kohmoto, Nana Okamoto, Takuya Naruto, Chie Murata, Yuya Ouchi, Naoko Fujita, Hidehito Inagaki, Shigeko Satomura, Nobuhiko Okamoto, Masako Saito, Kiyoshi Masuda, Hiroki Kurahashi, Issei Imoto

**Affiliations:** 1 Department of Human Genetics, Graduate School of Biomedical Sciences, Tokushima University, 3-18-15 Kuramoto-cho, Tokushima, 770-8503 Japan; 2 Department of Oral and Maxillofacial Surgery, Kobe University Graduate School of Medicine, 7-5-1 Kusunoki-cho, Chuo-ku, Kobe, Hyogo 650-0017 Japan; 3 Division of Molecular Genetics, Institute for Comprehensive Medical Science, Fujita Health University, 1-98 Dengakugakubo Kutsukake-cho, Toyoake, Aichi 470-1192 Japan; 4 Japanese Red Cross Tokushima Hinomine Rehabilitation Center for People with Disabilities, 4-1 Shinkai Chuden-cho, Komatsushima, Tokushima 773-0015 Japan; 5 Department of Medical Genetics, Osaka Medical Center and Research Institute for Maternal and Child Health, 840 Murodo-cho, Izumi, Osaka 594-1101 Japan

**Keywords:** 1q, Complex genomic rearrangement, Uniparental isodisomy, DUP-TRP/INV-DUP structure, Microhomology-mediated break-induced replication model, Template switching, Chromosomal microarray, Breakpoint junction sequence

## Abstract

**Background:**

Complex genomic rearrangements (CGRs) consisting of interstitial triplications in conjunction with uniparental isodisomy (isoUPD) have rarely been reported in patients with multiple congenital anomalies (MCA)/intellectual disability (ID). One-ended DNA break repair coupled with microhomology-mediated break-induced replication (MMBIR) has been recently proposed as a possible mechanism giving rise to interstitial copy number gains and distal isoUPD, although only a few cases providing supportive evidence in human congenital diseases with MCA have been documented.

**Case presentation:**

Here, we report on the chromosomal microarray (CMA)-based identification of the first known case with concurrent interstitial duplication at 1q42.12-q42.2 and triplication at 1q42.2-q43 followed by isoUPD for the remainder of chromosome 1q (at 1q43-qter). In distal 1q duplication/triplication overlapping with 1q42.12-q43, variable clinical features have been reported, and our 25-year-old patient with MCA/ID presented with some of these frequently described features. Further analyses including the precise mapping of breakpoint junctions within the CGR in a sequence level suggested that the CGR found in association with isoUPD in our case is a triplication with flanking duplications, characterized as a triplication with a particularly long duplication-inverted triplication-duplication (DUP-TRP/INV-DUP) structure. Because microhomology was observed in both junctions between the triplicated region and the flanking duplicated regions, our case provides supportive evidence for recently proposed replication-based mechanisms, such as MMBIR, underlying the formation of CGRs + isoUPD implicated in chromosomal disorders.

**Conclusions:**

To the best of our knowledge, this is the first case of CGRs + isoUPD observed in 1q and having DUP-TRP/INV-DUP structure with a long proximal duplication, which supports MMBIR-based model for genomic rearrangements. Molecular cytogenetic analyses using CMA containing single-nucleotide polymorphism probes with further analyses of the breakpoint junctions are recommended in cases suspected of having complex chromosomal abnormalities based on discrepancies between clinical and conventional cytogenetic findings.

**Electronic supplementary material:**

The online version of this article (doi:10.1186/s13039-017-0316-6) contains supplementary material, which is available to authorized users.

## Background

Complex genomic rearrangements (CGRs) consisting of two or more breakpoint junctions have been frequently observed during the characterization of nonrecurrent microduplications associated with genomic disorders [1, 2]. The occurrence of CGRs, such as partial tetrasomy induced by an interstitial triplication, contiguous distally with an extended segment uniparental isodisomy (isoUPD), has recently been reported as a rare event [3–7]. The recent establishment of high-resolution chromosomal microarray (CMA) using probes designed to detect copy number variations (CNVs) and genotype single-nucleotide polymorphism (SNP) simultaneously in a genome-wide manner has accelerated the identification of cases with such CGRs + isoUPD observations [8]. Although the cause, mechanism, and phenotypic effect of such CGR + isoUPD remain unclear, Carvalho et al. [5] provided evidence that CGRs generated post-zygotically through microhomology-mediated break-induced replication (MMBIR) can lead to regional isoUPD. In this replication-based mechanism model, a triplicated segment inserted in an inverted orientation between two copies of the duplicated segments (duplication-inverted triplication-duplication, DUP-TRP/INV-DUP) followed by regional isoUPD is generated via template switches between homologs and sister chromatids using MMBIR [5].

Here, we report on a patient with the co-occurrence of interstitial trisomy at 1q42.12-q42.2 and tetrasomy at 1q42.2-q43, followed by a segmental isoUPD for 1q43-qter, as additional evidence for an MMBIR-based model generating DUP-TRP/INV-DUP rearrangement followed by isoUPD. Detailed molecular genetic analyses at the sequence level revealed the presence of microhomology at two breakpoint junctions of the CGR, probably underlying the formation of the complicated genomic alteration (CGR + isoUPD). Notably, this is the first case of CGR + isoUPD detected in the long arm of chromosome 1. In addition, the pattern of flanking duplications experimentally documented in the present case, namely, a long duplicated segment with a size on the order of megabases at the centromeric junction observed by CMA with a short duplication at the telomeric junction only identified by sequencing of the breakpoint, has not been reported previously.

## Case presentation

The 25-year-old Japanese male reported on here was the first child of a non-consanguineous healthy mother (G0P0, 24 years of age) and father (details are unclear due to a divorce) with no notable family disease history. After an uncomplicated pregnancy, he had been born at 38 weeks of gestation by a normal delivery. His birth weight was 1958 g (−2.52 SD) and he was introduced into a neonatal incubator to treat intrauterine growth retardation (IUGR) and poor sucking by tube feeding for 20 days, although detailed medical records of his physique are not available. Physical examination at the age of 1 month showed height 46 cm (−3.4 SD), weight 2715 g (−2.6 SD), and head circumference 29.8 cm (−4.6 SD). The abilities to hold up his head, eat solid food, imitate the behaviors of others, and walk alone were recognized at 6 months, 18 months, 2 years and 6 months, and 3 years of age, respectively. The patient had never been able to speak until now, and his comprehension was limited to simple signs, but he recognized various sounds. At 3 years of age, he was diagnosed with the congenital heart defect of tetralogy of Fallot (TOF) but was not treated surgically, although he showed frequent squatting and cyanotic attacks. On physical examination at 24 years and 6 months of age, he showed growth retardation with height 136 cm (−6 SD), weight 28.1 kg (−3.3 SD), and severe mental retardation with a developmental quotient of 5. At 25 years of age, he had TOF, bilateral congenital inguinal hernia, bilateral cryptorchidism, club feet, scoliosis, Chilaiditi’s syndrome, and several facial anomalies, such as thinning of the hair, strabismus, widely spaced eyes, a down-slanted palpebral fissure, low-set ears, a prominent forehead, and a coarse face. He has some missing teeth due to having suffered from periodontal disease. Serial complete blood counts showed thrombocytopenia, and magnetic resonance imaging showed cerebral atrophy especially of the frontal lobe, with enlargement of the ventricles. His karyotype at birth was reported to be normal, but repeatedly performed karyotyping revealed 46,XY,dup(1)(q32.1q42.1),inv(9)(p12q13).

### Molecular cytogenetic studies

This research protocol for this study was approved by the local ethics committee of Tokushima University. Written informed consent for the participation of the patient in this study was obtained from the patient’s mother DNA was extracted from a peripheral blood sample.

**Fig. 1:**
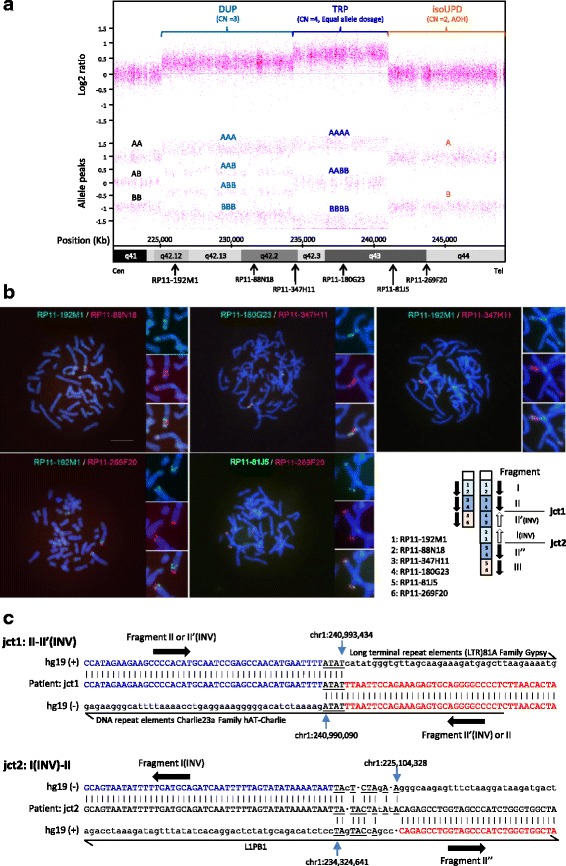
**a** Chromosome Analysis Suite (ChAS) graphic results of Affymetrix CytoScan HD analysis for the 1q region that presented duplication (DUP), triplication (TRP), or isoUPD in the patient. Detection of CGR and isoUPD were performed using an Affymetrix CytoScan HD CMA platform (Affymetrix), which provides 906,600 polymorphic (SNP) and 946,000 non-polymorphic (CNV) markers, according to the manufacturer’s recommendations. In addition, we used Chromosome Analysis Suite software (ChAS, Affymetrix) to process the raw data, and the output data were interpreted with the UCSC Genome Browser (http://genome.ucsc.edu; GRCh37/hg19 assembly). *Top*, copy number log2 ratio; bottom, allele peaks. CN, copy number. Possible genotype calls based on the allele dosage normalization algorithm are shown using A and B. The location of each BAC used for FISH analysis is shown. **b** Images of two-color FISH mapping using six BAC clones and the scheme of distal 1q CGR based on FISH data. Metaphase FISH images with high-magnification images of the distal 1q. BAC clones labeled with either FITC (*green*) or rhodamine (*red*) were hybridized to 4’,6-diamidino-2-phenylindole (DAPI)-stained chromosomes of the patient. The location and detailed information of each BAC are shown in Fig. 1a and Additional file 1: Table S1, respectively. In the scheme, arrows indicate the direction of chromosomal fragments I, II (II’, II”), and III, which presented duplication, triplication, and isoUPD, respectively, in CMA. Two junctions (jct 1 and jct2) between fragments II and II’ and between I and II” are also shown. **c** Color-matched sequence alignment of breakpoint junctions in rearrangements. Top, jct1 (breakpoint junction 1 between segments II and II’); *bottom*, jct2 (breakpoint junction 2 between segments I and II”) (see Fig. 1b). Microhomology at the junctions is represented by underlined letters. Frequent mismatch sequences were only observed near jct2 within long-range PCR products (data not shown). Thick arrows indicate the possible orientation of chromosomal fragments. Various types of repeat elements observed around junctions are shown

A high-resolution CMA using the CytoScan HD array (Affymetrix, Santa Clara, CA) with Chromosome Analysis Suite software (ChAS, Affymetrix) to process the raw data detected a 9.2-Mb trisomy at 1q42.12-q42.2, a 6.7-Mb tetrasomy consisting of the duplication of two haplotypes, each of which probably derives from either the father or the mother, at 1q42.2-q43, and a 8.2-Mb segment with the absence of heterozygosity at 1q43-qter consistent with isoUPD (arr[hg19]1q42.12q42.2(225,101,799_234,324,222)x3,1q42.2q43(234,330,738_240,992,219)x4,1q43qter(240,993,835_249,224,684)x2 hmz, Fig. 1a). Trisomic, tetrasomic, and iUPD regions contain 88, 38, and 94 Refseq genes, and 49, 21, and 24 OMIM genes, respectively. Neither copy number abnormalities nor iUPD around 1q42.2-qter was detected in the DNA of the patient’s mother (data not shown). Since the genotyping results using SNP typing probe within the iUPD region of the patient matched at least one of the maternal alleles, the iUPD segment is likely to have been inherited from his mother (data not shown), although genomic DNA of his father was not available to confirm the inheritance of this region. On the other hand, genotyping results within the trisomic region suggest that the duplicated segment is unlikely to have been inherited from his mother (data not shown). In the tetrasomic region (the triplicated segment), three allele peaks (AA, AB, and BB) with unusually large spaces between them were observed (Fig. 1a), suggesting the presence of AA/AA, AA/BB, and BB/BB tracks, which is only possible if each parent contributed equally with two alleles (either AA or BB).

Next, the location and orientation of each segment within this structurally altered region were determined by a series of dual-color fluorescence *in situ* hybridization (FISH) studies using bacterial artificial chromosome (BAC) clones located around the region (Fig. 1a and b, Additional file 1: Table S1) performed as described elsewhere [9]. Two signals (duplication) with a direct-inverted orientation and three signals (triplication) with a direct-inverted-direct orientation were detected by probes on the trisomic and tetrasomic regions, respectively. The triplicated segment in an inverted orientation was observed between the proximal triplicated segment in a direct orientation (junction 1, jct1) and the distal duplicated segment in an inverse orientation. The distal triplicated segment in a direct orientation is joined with the inversely oriented distal duplicated segment (junction 2, jct2). The isoUPD segment is then joined with this triplicated segment and terminates the abnormal chromosome 1. Taking these findings together, the final karyotype was interpreted as 46,XY,der(1)dup trp(pter → q43::q43 → q42.12::q42.2 → qter).

### Genomic investigation

For the precise mapping of breakpoint junctions in the CGR (jct 1 and 2), we first performed mate pair next-generation sequencing using the Nextera Mate Pair Sample Preparation Kit and Illumina HiSeq 1500 with 100 paired-end cycles according to the manufacturer instructions (Illumina, San Diego, CA). Reads were aligned to the human genome sequence using the Burrows-Wheeler Alignment tool 0.7.12. (http://bio-bwa.sourceforge.net). Two recurrent structural variations within 1q42.12-1qter were identified from the discordant read pairs around the estimated boundary areas by the expected number of reads per region and visual inspection using the Integrative Genomics Viewer. Long-range polymerase chain reaction (PCR) using primers designed around the estimated boundaries (Additional file 2: Table S2) and Takara LA Taq (Takara Bio, Otsu, Japan) with the two step protocol according to the manufacturer instructions. The direct sequencing of PCR products defined sequences around two breakpoint junctions, jct1 and jct2 (Fig. 1c). Based on these results, the duplication and the triplication start around chr1:225,104,328 and 234,324,641, respectively, and the triplication stops around 240,990,090. Interestingly, the small telomeric duplication, namely, of approximately 3 Kb, which evaded CMA detection, is located between 240,990,090 and 240,993,434, and isoUPD starts around 240,993,434, although the copy number of the distal flanking duplication was not experimentally validated. Therefore, the CGR observed in our case seems to involve triplication with flanking duplications, which has been characterized as a type II triplication proposed by Liu et al. [10] with a particular DUP-TRP/INV-DUP structure, and isoUPD was also reported to be associated with this type of CGR [5]. Notably, all reported cases with triplication with flanking duplications followed by isoUPD have small flanking duplications (<0.258 Mb and < 0.004 Mb in proximal and distal duplications, respectively) [5], indicating that our case is the first with a large proximal duplication (approximately 9.2 Mb) in this type of CGR. Microhomology (ATAT) was observed at the jct1 breakpoint interval, whereas a microhomologous sequence with some mismatch sequences including insertions, deletion, and point mutations was observed at the jct2 breakpoint interval (Fig. 1c). Mismatch sequences only near jct2 of CGR, which might occur during the same event as the *de novo* CGR/isoUPD formation, have previously been reported [5]. These mismatch sequences near to the breakpoint junctions of CGR are proposed to be one of the potential signature features of highly error prone replication-based mechanisms using DNA polymerase(s) of low fidelity or a replisome with reduced fidelity [2], although it remains unclear why mismatch sequences have been observed only in jct2 of CGR/isoUPD cases.

Within the isoUPD region, three genes were associated with four autosomal recessive diseases, as determined by a search of the Online Mendelian Inheritance in Man database (OMIM, http://www.omim.org, accessed 1 December, 2016; Additional file 3: Table S3). No phenotypes matching these four diseases were observed in the patient described here, and no pathogenic mutation was found in the three genes by Sanger sequencing. In addition, databases of imprinted genes, such as Geneimprint (http://www.geneimprint.com/site/genes-by-species, accessed 1 December, 2016) and the Catalogue of Parent of Origin Effects (http://igc.otago.ac.nz/home.html, accessed 1 December, 2016), indicated that there are no known imprinting genes within this isoUPD region.

## Discussion

**Fig. 2:**
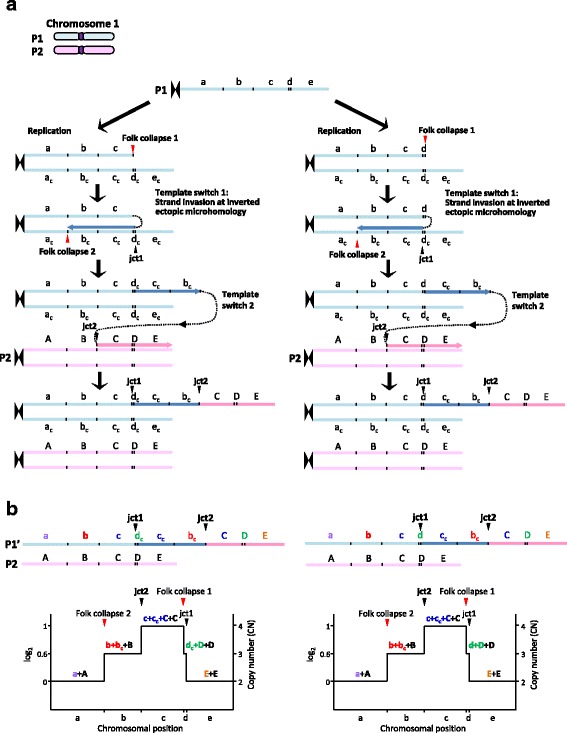
Replication-based mechanism model for the generation of DUP-TRP/INV-DUP rearrangement followed by isoUPD detected in the present case. **a** The event probably occurred involving parental homolog chromosomes, P1 and P2. The first template switch (template switch 1) have been triggered by a stalled or collapsed replication fork (fork collapse 1), and used a complementary strand to resume replication through using microhomology in the complementary strand at the annealing site (jct1, Fig. 1c) to prime DNA synthesis, resulting in the production of a segment with the inverse orientation compared with the reference genome. Two putative jct1 sites, jct1 between c and d_c_ (*left*) and jct1 between d and c_c_, (*right*) are predicted, because the same sequence result can be obtained in both cases (see Fig. 1c). Then, a new fork stalling or collapsing event (fork collapse 2) have released a free 3’ end that can be resolved by the second template switching (template switch 2) through using the microhomology in the homologous chromosome at the annealing site (jct2, Fig. 1c) to prime and resume DNA synthesis, resulting in the generation of jct2 as well as isoUPD. a–d, representative chromosome alleles in P1 chromosome; a_c_–e_c_, complementary chromosome alleles in P1 chromosome; A–E: corresponding homologous chromosome alleles in the P2 chromosome. **b** *Top*: different genomic structures are predicted to be generated depending on the location of the selected annealing site (jct1) to prime DNA synthesis in the first template switch event. isoUPD will result if the unidirectional replication fork continues until the telomere. *Bottom*: predicted segmental CNV in a simulated CMA. Note that the small size of the telomeric duplication between fork collapse 1 and jct1 led to the evasion of CMA detection (Fig. 1a), because the region was too small to be detected by Affymetrix Cytoscan HD array

In the case presented here, our comprehensive analyses of all of the cytogenetic, microarray, and sequencing data suggest that the MMBIR-based template-switching model (Fig. 2a) recently proposed by Carbalho et al. [5] is one of the most plausible mechanisms underlying the gain of interstitial copy number followed by distal isoUPD to the telomere, which has not previously been described in the long arm of chromosome 1. In this model, two-step template switches triggered by stalled or collapsed replication forks might have occurred. The first template switch is supposed to use a sister chromatid to resume replication. Microhomology at the annealing site (jct1, Fig. 1c) in the complementary strand close to breakpoint is used to prime DNA synthesis, although it is difficult to determine whether this template switching occurred between c and d_c_ or d and c_c_ in our sequencing method. Then, unidirectional replication resumes in an inverted orientation and forms an inverted partially duplicated segment. A new event of fork stalling or collapsing might occur and release a free 3’ end, which can be resolved by a second template switch to the homologous chromosome using microhomology again, resulting in the formation of a jct2 (Figs. 1c and 2a). This second compensating inversion might contribute to result in a viable cell. A target annealing site was selected between alleles B and C in the present case, and the derivative chromosome results in a DUP-TRP/INV-DUP structure with a unique long proximal duplicated region (b and b_c_, Fig. 2b). Because BIR cannot account for the observations of microhomology identified in both jct1 and jct2 (Fig. 1c), MMBIR is probably involved in resolving both the first and the second breaks. In our case and some previously reported cases [5], however, various mismatch sequences including insertions, deletions, and/or point mutations around breakpoint junction sequences were observed only in jct2 of CGR and the size of the proximal duplicated region containing jct2 was commonly larger than that of the distal duplicated region containing jct1. Therefore, the accomplishment of the resolution of the second break might need additional mechanisms. It also remains unknown whether those two events occurred either all at once in a post-zygotic mitotic cell or in two steps: the first step occurring in a pre-meiotic cell was resolved by the second step occurring in a post-zygotic cell. These alternatives cannot be distinguished using the current data. In addition, it also difficult to rule out tissue-specific mosaicism as a post-fertilization mitotic event in this case, although no finding of mosaicism was observed in all data obtained from the peripheral leukocytes/lymphocytes of the patient.

Recently, several cases along with our own with concurrent triplication (tetrasomy) and isoUPD, which may be explained by the MMBIR-based mechanism, detected by CMA containing SNP probes, have been reported [4–7]. However, detailed analyses of centromeric and telomeric junctions of triplicated regions in a tiling array or at the sequence level have only been performed on the cases reported by Carvalho et al. [5] and the present case. In most of those cases with detailed junctional analyses, relatively short flanking duplications were observed. These findings suggest that the small size of flanking duplications might have led to the evasion of array-based detection in three reported cases without detailed junction analyses [4, 6, 7]. Indeed, the concurrent triplication (tetrasomy) and isoUPD were detected by Affymetrix arrays including SNP probes in all cases, but a flanking duplication was observed in this analysis only at the centromeric junction in the present case. In addition, microhomology was observed in breakpoint junctions in most of the cases with the DUP-TRP/INV-DUP rearrangement followed by isoUPD reported by Carvalho et al. [5] and the present case, suggesting that an MMBIR-based mechanism might underline the formation of at least this type of genomic alteration implicated in constitutional disorders. Detailed junction analyses of additional cases showing CGRs + isoUPD will be needed to provide support for an MMBIR-based mechanism inducing complex copy number gains and segmental isoUPD in tandem in subjects with multiple congenital anomalies.

Because partial 1q trisomy is a rare disorder and unbalanced chromosomal translocations are often observed with this alteration [11–16], it is difficult to evaluate the contribution of 1q trisomy to the phenotype in cases involving another chromosome. Patients with pure partial distal trisomy 1q are known to demonstrate a wide range of manifestations of variable severity. However, distal 1q duplication syndrome is characterized by the signs present in many of the previously reported cases [15, 16]. The present case showed some of the symptoms characteristic of distal 1q duplication syndrome, such as psychomotor developmental delay, cardiac defect, widely spaced eyes, a down-slanted palpebral fissure, low-set ear, a prominent forehead, club feet, and scoliosis, although psychomotor developmental delay and cardiac defect were very severe compared with those in previously reported cases and some features commonly found elsewhere were not observed [15, 16]. Because the present patient is the first known case of pure distal partial 1q tetrasomy and trisomy, it is possible that the copy number increase in some of the genes located between 1q42.12 and the middle of 1q43 (approximately 180 RefSeq genes) contributes to these symptoms, although no causal regions responsible for each symptom of distal trisomy/tetrasomy 1 syndrome have been clarified. In addition, the influence of isoUPD on the clinical features of the present case remains unknown because of a lack of reported cases with distal 1q UPD.

## Conclusions

We report the first case with concurrent CGR (duplications and triplication) + isoUPD in 1q42.12-qter, from an initial diagnosis of interstitial trisomy 1q by conventional karyotyping. Comprehensive cytogenetic and molecular analyses provide additional evidence that DUP-TRP/INV-DUP rearrangement having a unique long proximal DUP structure followed by isoUPD may be generated by an MMBIR-based mechanism. Because it is almost impossible to quantify precise chromosomal copy numbers and detect UPD by conventional karyotyping, molecular cytogenetic analyses using CMA containing SNP probes with additional detailed analyses of the breakpoint junctions in a sequence level are recommended in cases suspected of having complex chromosomal abnormalities based on clinical and cytogenetic findings.

## Additional files

Additional file 1: Table S1. BAC clones used in FISH experiments. (DOCX 14 kb)

Additional file 2: Table S2. List of primer sets used in PCR and sequencing for junctions of the CGR. (DOCX 14 kb)

Additional file 3: Table S3. Autosomal recessive diseases and causative genes aound the isoUPD region. (DOCX 14 kb)

## Abbreviations

BAC: Bacterial artificial chromosome
CGR: Complex genomic rearrangements
CMA: Chromosomal microarray
CNV: Copy number variation
DUP-TRP/INV-DUP: Duplication-inverted triplication-duplication
FISH: Fluorescence *in situ* hybridization
isoUPD: Uniparental isodisomy
IUGR: Intrauterine growth retardation
MCA: Multiple congenital anomalie
MMBIR: Microhomology-mediated break-induced replication
PCR: Polymerase chain reaction
SNP: Single-nucleotide polymorphism
TOF: Tetralogy of Fallot
