# Supplementary material for: A case with concurrent duplication, triplication, and uniparental isodisomy at 1q42.12-qter supporting microhomology-mediated break-induced replication model for replicative rearrangements
Source: Mol Cytogenet. 2017 Apr 28;10:15. doi: 10.1186/s13039-017-0316-6 (PMC5410019; doi:10.1186/s13039-017-0316-6)
Supplement: Supplementary file 1 [file 13039_2017_316_MOESM1_ESM.docx]

Additional file 1: Table S1. BAC clones used in FISH experiments

No.	BAC ID	Location (according to hg19)	Relative position within altered regions on 1q	
1	RP11-192M1	chr1:226,055,092-226,210,301	Trisomic	proximal
2	RP11-88N18	chr1:231,727,974-231,884,507		distal
3	RP11-347H11	chr1:234,525,172-234,686,386	Tetrasomic	proximal
4	RP11-180G23	chr1:237,835,740-238,000,038		distal
5	RP11-81J5	chr1:241,346,802-241,525,078	isoUPD[a]	proximal
6	RP11-269F20	chr1:243,708,834-243,711,631		distal

[a]isoUPD, uniparental isodisomy
