# Supplementary material for: A case with concurrent duplication, triplication, and uniparental isodisomy at 1q42.12-qter supporting microhomology-mediated break-induced replication model for replicative rearrangements
Source: Mol Cytogenet. 2017 Apr 28;10:15. doi: 10.1186/s13039-017-0316-6 (PMC5410019; doi:10.1186/s13039-017-0316-6)
Supplement: Supplementary file 2 [file 13039_2017_316_MOESM2_ESM.docx]

Additional file 2: Table S2. List of primer sets used in PCR and sequencing for junctions of the CGR

Primer name	Sequence
jct2 (junction between I and II) PCR and sequencing primers	
A-R4	5'-GTACTACCACCTTGTTCAACC-3'
B-R3	5'-GCTGAGACTTTCCAGTACATC-3'
jct1 (junction between II and II') PCR primer[a]	
C-F2	5'-GCCTTCTAGTTGTACGTGTTG-3'
jct1 sequencing primer	
C-F8	5'-GTTTCTTCATGACCTGGAGG-3'

[a]only one primer was used for PCR
