# Supplementary material for: A case with concurrent duplication, triplication, and uniparental isodisomy at 1q42.12-qter supporting microhomology-mediated break-induced replication model for replicative rearrangements
Source: Mol Cytogenet. 2017 Apr 28;10:15. doi: 10.1186/s13039-017-0316-6 (PMC5410019; doi:10.1186/s13039-017-0316-6)
Supplement: Supplementary file 3 [file 13039_2017_316_MOESM3_ESM.docx]

Additional file 3: Table S3. Autosomal recessive diseases and causative genes aound the isoUPD region[a,b]

Location (hg19)	Cytoband	Gene	Gene MIM number	Phenotype	Phenotype MIM number
chr1:241,660,856	1q43	*FH*	136850	Fumarase deficiency	606812
chr1:243,419,306	1q43	*SDCCAG8*	613524	Senior-Loken syndrome 7	613615
				Bardet-Biedl syndrome 16	615993
chr1:244,998,607	1q44	*COX20*	614698	Mitochondrial complex IV deficiency	220110

[a]The list was created according to OMIM (http://www.omim.org) on 1 December 2016)

[b]chr1: 240,993,835- 249,224,684 according to hg19 detected by Affymetrix CytoScan HD array
